# RTKN2 knockdown alleviates the malignancy of breast cancer cells by regulating the Wnt/β-catenin pathway

**DOI:** 10.1038/s41598-023-50153-w

**Published:** 2023-12-27

**Authors:** Xiaomei Zhang, Jian Wang, Haiying Li

**Affiliations:** 1 Department of Radiotherapy, Qilu Hospital of Shandong University, Jinan, 250012 Shandong China; 2 Department of Ultrasound, Shandong Province Coal Taishan Sanatorium, Taian, 271000 Shandong China; 3 Department of Ultrasound, Qilu Hospital of Shandong Univesity, No. 107, Wenhuaxi Rd., Jinan, 250012 Shandong China

**Keywords:** Cancer, Cell biology, Oncology

## Abstract

RTKN2 is a new effector protein of Rho GTPase, and has been indicated to be a tumor inhibitor in colon cancer. In this article, we explored the function of RTKN2 in BC cell development. RTKN2 expression in BC tissues and BC cell lines was evaluated by RT-qPCR and Western blot assay. CCK-8, Wound-healing and Transwell assays were carried out to examine the role of RTKN2 knockdown on proliferation, the migratory ability and the invasive ability of BC cells. FCM and Western blot assay were performed to measure the function of RTKN2 silencing on BC cell apoptosis. In addition, the regulatory effect of RTKN2 on Wnt/β-catenin pathway was studied via Western blot assay. RTKN2 expression was elevated in BC tissues and BC cells. Down-regulation of RTKN2 restrained BC cell progression by suppressing cell proliferation, migratory ability, invasive ability, and inducing apoptosis. In addition, reduced of RTKN2 sharply reduced the expressing levels of Wnt3A, β-catenin, C-Myc, and Cyclin D1, suggesting that RTKN2 silencing blocked the motivation of Wnt/β-catenin pathway in BC development. The in vivo experiment also confirmed the inhibitory effect of RTKN2 on BC tumors. Our study confirmed that RTKN2 was highly expressed in BC. Moreover, RTKN2 knockdown suppressed the development of BC through affecting the Wnt/β-catenin pathway. Hence, we deduced that RTKN2 was a possible treatment target for BC.

## Introduction

Breast cancer (BC) is the commonest and deadliest tumor among women in more than 100 countries^1^. In 2020, there will be nearly 2.3 million new cases of BC worldwide^2^. BC is a kind of much factor disease. Factors such as genetics, environmental factors, dietary habits, and mental stress can all induce BC^3,4^. BC screening can effectively increase the early diagnosis rate and prolong the survival time^5^. Additionally, tumor metastasis and rapid growth accelerate the death process of BC patients^6^. In consequence, research on the molecular pathogenesis of BC should be strengthened to provide scientific support for the prevention and treatment of BC.

Rho GTPase family can maintain cell polarity, cytoskeletal structure, cell–matrix adhesion, and take part in the regulation of cell transformation^7^. In recent years, Rho GTPase family has been reported to be participated in the regulation of tumor cell proliferation, apoptosis and differentiation^8,9^. The downstream proteins of Rho GTPase related to tumor regulation mainly include ROCK, PAK, Rhotekin (RTKN), etc.^10^. Qu et al*.* confirmed that reduction of RTKN hampered the occurrence of colon cancer by suppressing cell growth and metastasis^11^. Moreover, RTKN acted as an oncogenic gene in gastric cancer through the regulation of p53 and HDAC1^12^. RTKN2 is a newly discovered RTKN protein, which locates at 10q21.2^13^. It’s been reported that RTKN2 silencing blocked the growth and accelerated the apoptosis in ovarian cancer cells^14^. RTKN2 knockdown clearly inhibited the migration and proliferation of BC cells^15^. However, other molecular mechanisms of action of RTKN2 in breast cancer remain unknown.

As one of the most studied Wnt signaling pathways, Wnt/β-catenin pathway plays a crucial part in early embryo development, organogenesis, tissue regeneration and other physiological processes^16^. Furthermore, the abnormalities of Wnt/β-catenin pathway have been involved in the development of multiple human diseases^17,18^. A large number of reports have confirmed that Wnt/β-catenin pathway is related to the genesis and development of BC. CBX7 blocks the Wnt/β-catenin pathway by enhancing Wnt antagonist DKK-1, thereby inhibiting the development of BC^19^. The study on Wnt/β-catenin pathway not only contributes to understanding the pathogenesis of human diseases, but also provides a series of effective targets for the treatment of diseases.

In this paper, RTKN2 expression in BC tissues and cells was firstly explored. Additionally, we revealed the role of RTKN2 knockout on cell growth, invasion, migration and apoptosis of BC cells. Additionally, we investigated whether RTKN2 knockout can affect the Wnt/β-catenin pathway in BC cell development.

## Materials and methods

### Tissue samples

Tissue specimens and adjacent non-cancer specimens were obtained from 24 BC patients in our hospital from December 2020 to October 2021. The patients were not received any treatment before enrollment and were diagnosed with BC by pathological diagnosis. This research was approved by the Ethics Committee of Qilu Hospital of Shandong University (approval number: KYLL-202011-048). All patients in the study were informed and provided the written informed consent. All procedures and methods were performed following the relevant guidelines and regulations, including the Declaration of Helsinki.

### Cell clines and cell transfection

BC cell lines BT549, MDA-MB-231, MDA-MB-468 and normal human mammary epithelial cells MCF-10A were provided by the gynecological oncology laboratory of our hospital. The cells were grown in RPMI 1640 medium with 10% FBS, 100 U/mL penicillin and 100 mg/mL streptomycin. All cells were cultivated at 37 °C and 5% CO_2_.

Transfection experiment was performed when the cell density was 70%. The transfection agent (Lipofectamine2000, Invitrogen, USA) and si-RTKN2 (siRTKN2-1: GCAGGACTGCAACATTCAAGA, siRTKN2-2: GCTCGACTAATGGCCTATACA, Gemma, Shanghai, China) were added to BT549 and MDA-MB-231cells. After 48 h of culture, Western blot assay was carried out to evaluate the transfection efficiency of RTKN2 knockdown.

### RT-qPCR

TRIzol kit was applied to isolate the total RNA from BC cells and BC tissues. The total RNA was then reversely transcribed into cDNA via the Promega kit (Toyobo, Japan). Then, cDNA was used as raw material for PCR amplification. PCR reaction system was: 1μL cDNA, 6.25μL SYBR, 0.25μL ROX, 0.5μL upstream primers, 0.5μL downstream primers and 4μL DEPC water. GAPDH was the internal reference of RTKN2. 2^−△△CT^ method was performed to analyze the relative level of RTKN2. The sequences of primers are as follows: RTKN2 Forward, 5’-ATGCTCGACTAATGGCCTATACA-3’; RTKN2R Reverse, 5’-CGTCGTGATCGTTCTTTATTGCT-3’; GAPDH Forward, 5’- GATTCCACCCATGGCAATTC-3’; GAPDH Reverse, 5’- AGCATCGCCCCACTTGATT-3’.

### Western blot assay

The cells of each group were mixed with cell lysates on ice to obtain the total protein. After SDS-PAGE gel electrophoresis, the total protein was transferred to PVDF membrane. After sealed with 50 g/L skim milk powder for 2 h, the primary antibody (anti-RTKN2, 1:1000, Proteintech; anti-Bax, 1:2000, Abcam; anti-Bcl-2, 1:1000, Abcam; anti-Wnt3A, 1:1000, ABclonal; anti-c-myc, 1:1000, Proteintech; anti-cyclin D1, 1:1000, Proteintech; anti-beta catenin, 1:1000, Proteintech; anti-beta actin, 1:5000, Proteintech) was incubated at 4 °C overnight. After washing with TBST for 3 times, the second antibody was incubated for 2 h. ECL luminescent solution was used to develop the target strip.

### CCK-8 assay

The cells of logarithmic growth stage were digested and then inoculated in a 96-well plate. After 0,24,48,72 h, 10 µl of CCK-8 solution (C0037, Beyotime, China) was added to each well of the plate. Then the plate was incubated for 1 h at 37 °C. Cell proliferation was determined by scanning with a microplate reader at 450 nm.

### Wound-healing assay

The cells at logarithmic growth stage were digested and made into single cell suspension at the density of 2 × 10^6^/mL. 2 mL cell suspension was added into each 6-well plate well. On the second day, a 10uL tip was scratched perpendicular to the cell plane. Photos were taken at 0 and 24 h for subsequent data analysis.

### Transwell assay

100μL Matrigel gel (1:8, BD, USA) was added to the upper chamber of Transwell chamber (0.8 μm). 500μL complete medium was added to the lower chamber. 100μL single cell suspension was cultured in the upper chamber for 48 h. After washing off the non-adherent cells, the cells were fixed with paraformaldehyde (4%), and stained with crystal violet (0.1%). After cleaning and drying, the cells were photographed via a microscope.

### Flow cytometry (FCM)

The cells were washed with pre-cooled PBS, digested with trypsin. After centrifugation, cells were added with FITC-labeled Annexin V and Binding Buffer. Then, 5 μL Propidium Iodide was added at room temperature away from light. After 5–15 min, FCM was performed to test cell apoptosis.

### In vivo experiment

5-week-old BALB/C nude mice were obtained from Cyagen (Suzhou, China). The nude mice were fed under constant temperature, humidity and aseptic conditions. 200μL BT549 cells with si-RTKN2 were injected into nude mice subcutaneously. After inoculation, the tumors of nude mice were observed every 4 days. After 4 weeks, the mice were sacrificed, and subcutaneous tumors were isolated to weigh and photograph for preservation. Experiments were conducted in compliance with National Institutes of Health guidelines and followed procedures approved by the Qilu Hospital of Shandong University Animal Care and Use Committee. All studies reported herein were performed in accordance with ARRIVE guidelines (https://arriveguidelines.org).

### Statistical analysis

The data were statistically analyzed by SPSS 22.0. The data were expressed as mean ± SD. The difference between the two groups was analyzed by *t* test. All experiments were repeated at least three times. The difference was statistically significant when p < 0.05.

## Results

### Enforced expression of RTKN2 was in BC

**Figure 1:**
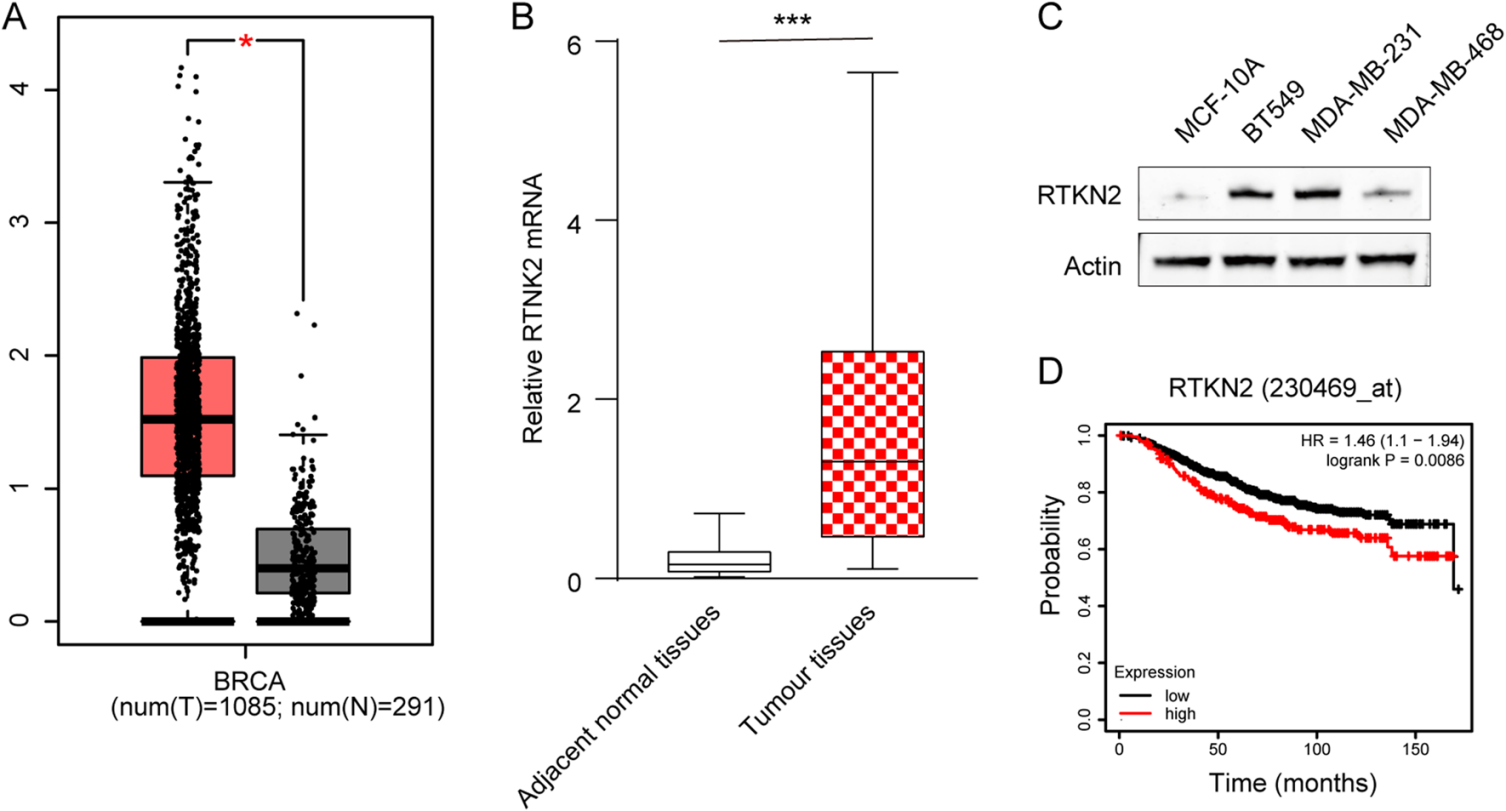
Enforced expression of RTKN2 was in BC. (**A**) The expression of RTKN2 in BC tissues in GEPIA database. (**B**) The expression of RTKN2 in 24 BC tissues form our hospital was detected by RT-qPCR. (**C**) The expression of RTKN2 in MCF-10A, MDA-MB-231, MDA-MB-468 and BT549 cells. (**D**) Kaplan–meier analysis showed an association between RTKN2 expression and overall survival rate in BC patients. *p < 0.05, ***p < 0.001.

Initially, we searched the expression pattern of RTKN2 in BC via GEPIA database. Compared with 291 normal tissues, RTKN2 was clearly upregulated in most of 1085 BC tissues (Fig. 1A). Subsequently, enforced expression of RTKN2 was also validated in 24 BC samples from our hospital (Fig. 1B). Meanwhile, RTKN2 in BC cell lines was higher than in MCF-10A cells (Fig. 1C). Kaplan–Meier analysis demonstrated that the patients with elevated RTKN2 had dramatically lower overall survival rate than that of patients with reduced RTKN2 (Fig. 1D). Hence, RTKN2 was overexpressed in BC and was associated with the poor prognosis of BC patients.

### RTKN2 silencing hampered cell proliferation, the migratory ability and the invasive ability of BC cells

**Figure 2:**
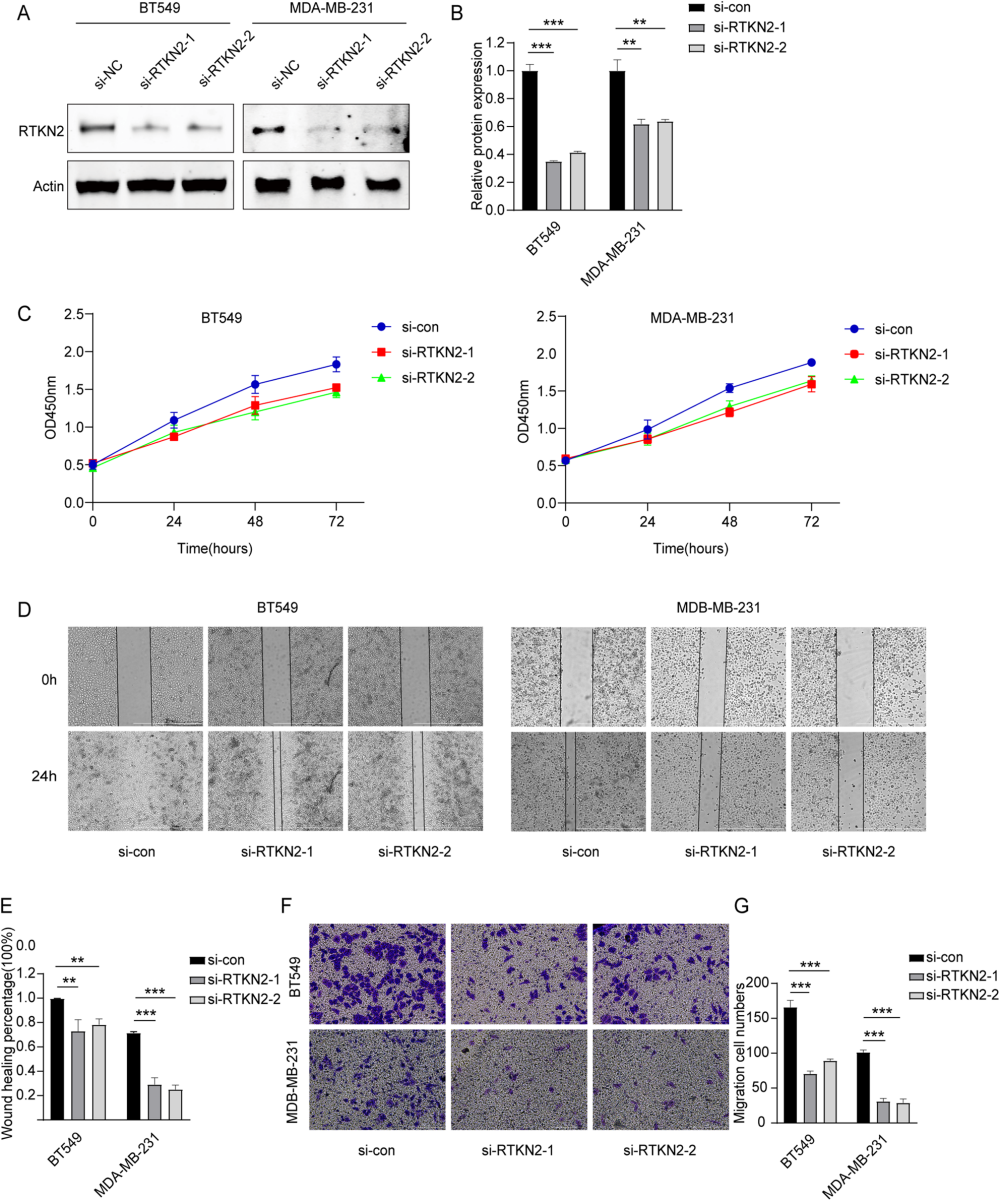
RTKN2 silencing hampered cell proliferation, the migratory ability and the invasive ability of BC cells. (**A**,**B**) The transfection rate of RTKN2 knockdown. (**C**) Cell proliferation of BT549 and MDA-MB-231 cells with RTKN2 knockdown was measured by CCK-8 assay. (**D**,**E**) The migratory ability was measured by Wound-healing assay. (**F**,**G**) The invasive ability was detected by Transwell assay. **p < 0.01, ***p < 0.001.

To ascertain the function of RTKN2 in BC progression, we knocked down the expression of RTKN2 in BT549 and MDA-MB-231cells. The successful transfection of RTKN2 was verified by Western blot assay (Fig. 2A,B). Results of CCK-8 assay displayed that RTKN2 silencing reduced cell proliferation in BT549 and MDA-MB-231 cells (Fig. 2C). Additionally, the migratory ability was inhibited in BT549 and MDA-MB-231 cells transfected with RTKN2 knockdown by Wound-healing assay (Fig. 2D,E). Also, the invasive ability was attenuated by RTKN2 knockdown (Fig. 2F,G). All these data confirmed that RTKN2 knockdown played a tumor suppressor in BC cell progression.

### RTKN2 knockdown promoted cell apoptosis

**Figure 3:**
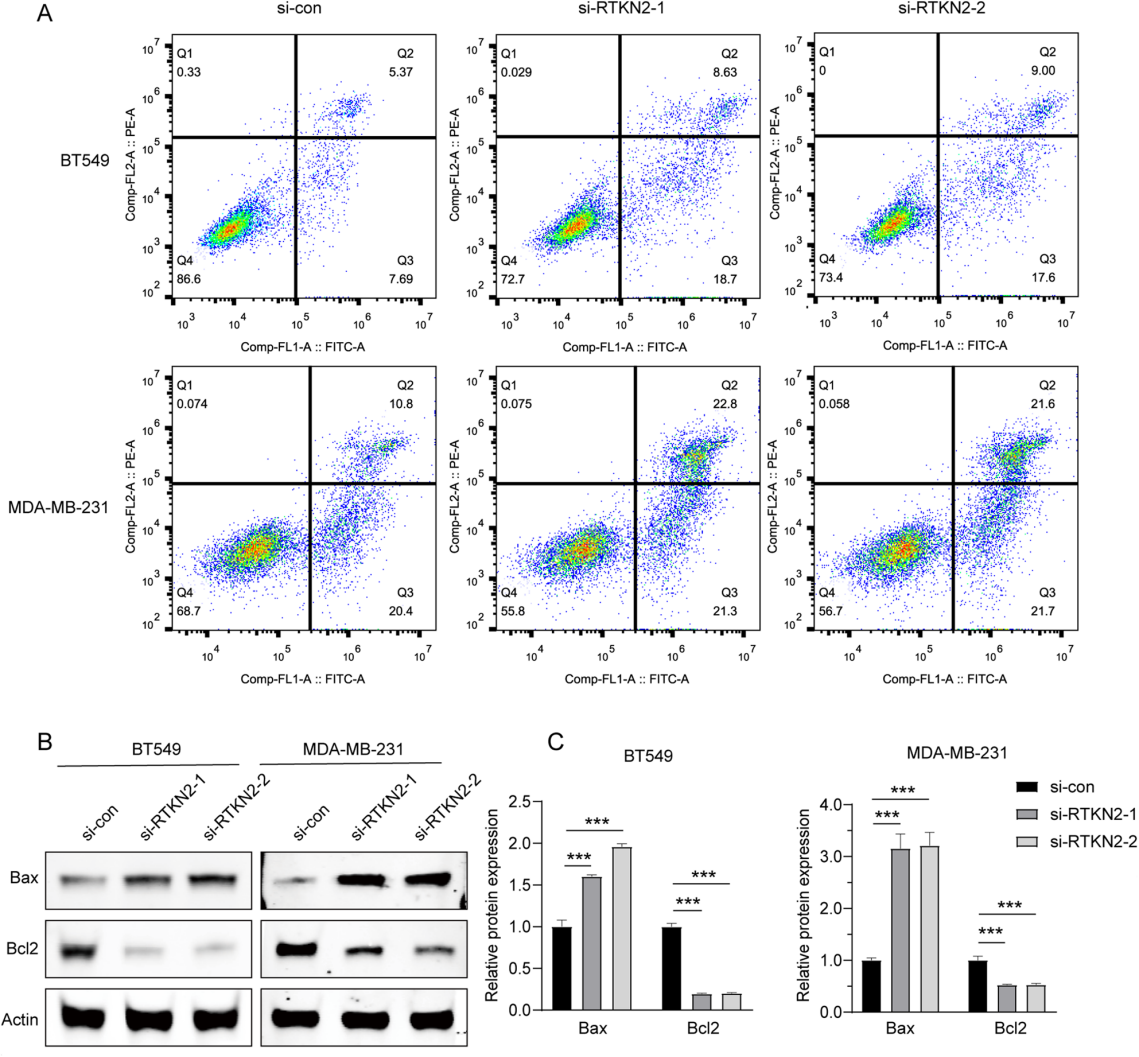
RTKN2 knockdown promoted cell apoptosis. (**A**) Cell apoptosis of BT549 and MDA-MB-231 cells with RTKN2 knockdown was detected by FCM. (**B**,**C**) The expression of Bcl-2 and Bax were detected by Western blot assay. **p < 0.01, ***p < 0.001.

Furthermore, cell apoptosis was evaluated by FCM and the protein expression of Bcl-2 and Bax. The results of FCM analysis showed that RTKN2 knockdown group had more apoptotic population than si-NC group (Fig. 3A). The expression of Bcl-2 was reduced, and the expression of Bax notably elevated in BT549 and MDA-MB-231 cells transfected with RTKN2 knockdown (Fig. 3B,C). All findings indicated that the reduced expression of RTKN2 facilitated apoptosis of BC cells.

### RTKN2 regulated Wnt/β-catenin pathway in BC cells

**Figure 4:**
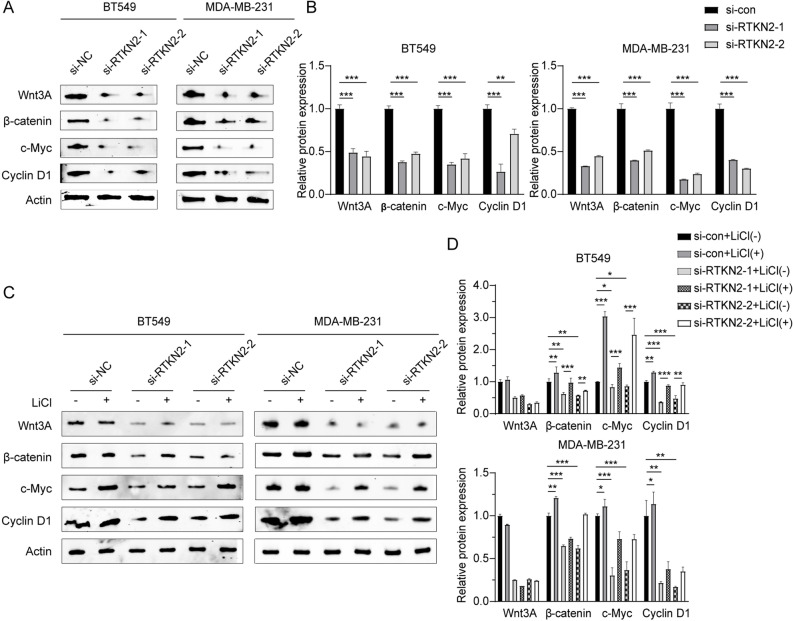
RTKN2 regulated Wnt/β-catenin pathway in BC cells. (**A**,**B**) The expression of Wnt3A, β-catenin, C-Myc, and Cyclin D1 were detected by Western blot assay. (**C**,**D**) The expression of Wnt3A, β-catenin, C-Myc, and Cyclin D1 were detected after treated with LiCl or RTKN2 knockdown. *p < 0.05, **p < 0.01, ***p < 0.001.

Western blot assay was carried out to measure the influence of RTKN2 knockdown on Wnt/β-catenin pathway related proteins in BT549 and MDA-MB-231 cells. We found thatWnt3A, β-catenin, C-Myc, and Cyclin D1expression were sharply reduced after RTKN2 knockdown, and the activity of Wnt/β-catenin pathway was inhibited (Fig. 4A,B). As an inhibitor of GSK3β, LiCl can directly activate β-catenin across Wnt, thus activating the Wnt/β-catenin pathway. Next, cells transfected with RTKN2 silencing were treated Wnt/β-catenin pathway agonist LiCl. The presence or absence of LiCl did not change the effect of RTKN2 knockout on Wnt3A expresison. Moreover, LiCl reversed the down-regulation effect of RTKN2 knockout on β-catenin, C-Myc, and Cyclin D1, but the expression levels of β-catenin, C-Myc, and Cyclin D1 were still lower compared with the si-NC group. (Fig. 4C,D). These data indicated that RTKN2 knockdown blocked the stimulation of Wnt/β-catenin pathway in BC progression.

### RTKN2 silencing repressed tumor growth in vivo

**Figure 5:**
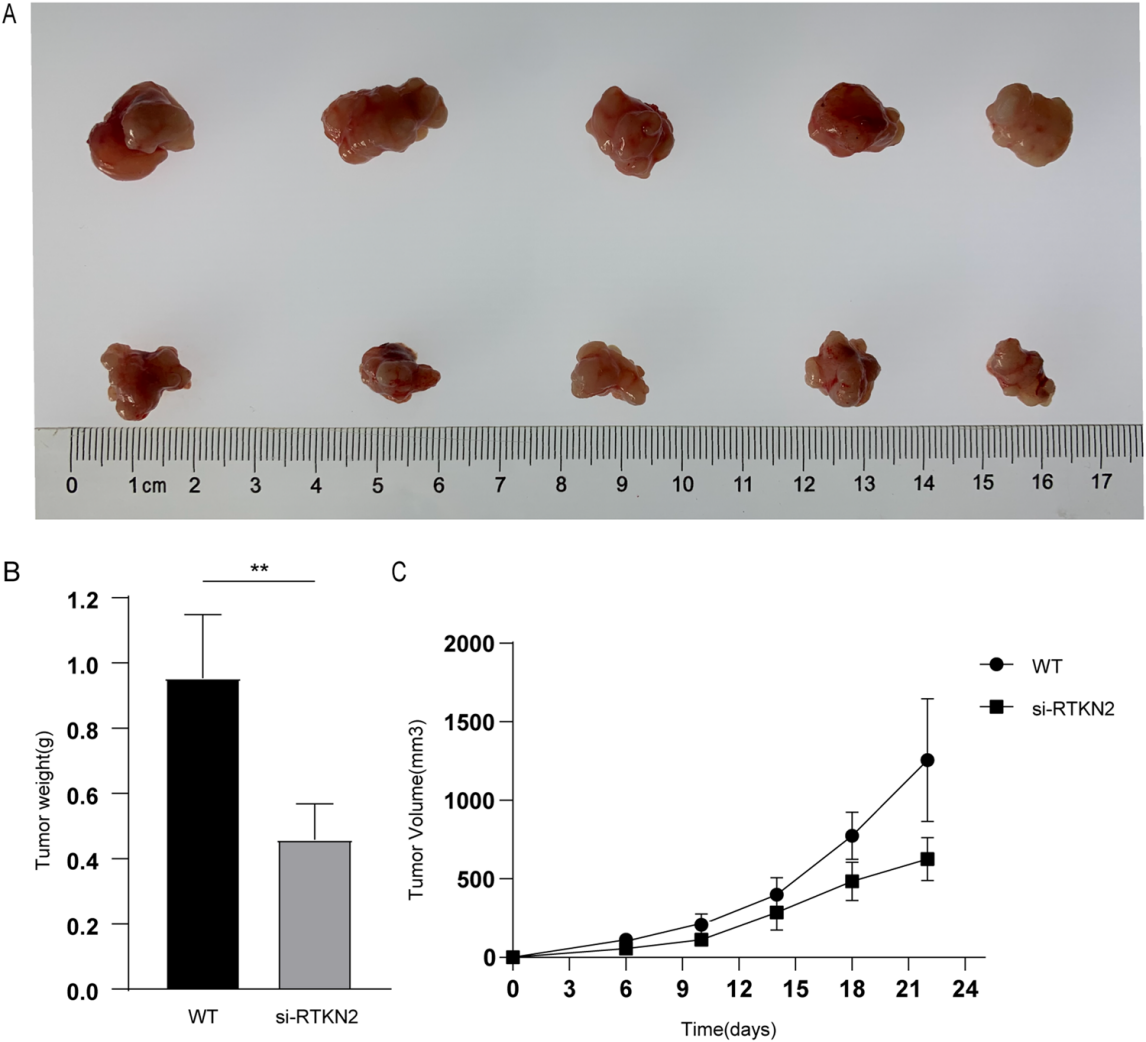
RTKN2 silencing suppressed tumor growth in vivo. (**A**) Representative images of xenograft tumors. (**B**) After 4 weeks, the tumor weight was measured. (**C**) The tumor volume was measured every 4 days for 4 weeks. **p < 0.01.

To further investigate the tumogenic effect of RTKN2 on BC in vivo, BT549 cells with sh-RTKN2 were injected into 5-week-old nude mice. As shown in Fig. 5A,C, the weight and volume of tumors were notably smaller in the si-RTKN2 group than the si-NC group. Our data indicated that RTKN2 silencing blocked tumor progression in vivo.

## Discussion

The occurrence of BC may be related to the activation of oncogenes, inactivation of tumor suppressor genes, abnormal cell cycle regulation and changes in the level of pathway-related proteins, which is a complicated process involves various genes and signaling pathways^20,21^. RTKN2 is a new effector protein of Rho GTPase family, and has been identified to serve as a specific role in several cancers. RTKN2 was upregulated in hepatocellular carcinoma, and enhanced expression of RTKN2 suppressed cell apoptosis and facilitated the migration and invasion^22^. Conversely, overexpression of RTKN2 blocked the glycolysis, cell growth, invasion and migration of lung cancer cells via the regulation of the NF‑κB pathway^23^. In our paper, enforced expression of RTKN2 was found in BC. It is worth noting that the effect of RTKN2 silencing on BC cell proliferation, the migratory ability, the invasive ability and cell apoptosis was investigated. Our data confirmed that RTKN2 knockdown remarkably slowed the progression of BC cells, which was consistent with Wu’s findings^15^. Additionally, we also demonstrated that RTKN2 knockdown restrained tumor growth in vivo. Similarly, RTKN2 exacerbated the tumor development by regulating MM92 and MMP9 expression in non-small cell lung cancer^24^. Moreover, reduced of RTKN2 blocked cell invasion, cell growth and promoted cell apoptosis in hepatocellular carcinoma^25^. Thus, our results showed that RTKN2 is a cancer-promoting factor in BC.

Wnt/β-catenin pathway mainly takes part in cell proliferation, apoptosis, polarization, and differentiation processes^26^. When β-catenin content reaches a certain level, it can enter the nucleus from cytoplasm and accelerate the metastasis of tumor cells^27^. BMX activated the Wnt/β-catenin pathway by increasing β-catenin, p-β-catenin (Y654), p-β-catenin (S33/37), p-β-catenin (Y142),c-myc and p-GSK2β (S9)^28^. TMED3 leaded to the stimulation of Wnt/β-catenin signaling pathway by increasing β-catenin, cyclin D1, Axin2, MMP7, c-myc and TCF4 in BC cells^29^. In our paper, we investigated how RTKN2 down-regulation acts on the Wnt/β-catenin pathway in BC cells. Consistent with previous study, RTKN2 knockdown sharply reduced Wnt3A, β-catenin, C-Myc, and Cyclin D1. Furthermore, RTKN2 silencing decreased C-Myc, PCNA and Cyclin D1 expression levels in colon cancer^30^.

Besides that, we list several shortcomings of this study. First, we studied a relatively small number of tissue samples, which may reduce statistical accuracy. Secondly, other experiments should be used to verify our results, such as clone formation assay. Therefore, further experiments are needed to verify our conclusions.

## Conclusion

In sum, RTKN2 was identified as a highly expressed gene in BC. Furthermore, RTKN2 knockdown blocked the development of BC cells by repressing Wnt/β-catenin pathway. Therefore, RTKN2 may be a possible biomarker target for BC therapy.

## Data Availability

The datasets during the current study are available from the corresponding author on reasonable request.
